# Outcome of Operated Colorectal Cancers in Relation to the Type of Initial Referral

**DOI:** 10.7759/cureus.36840

**Published:** 2023-03-29

**Authors:** Mahmoud Elnaggar, Ponnuthurai Pratheepan, Baskaran Paramagurunathan, Josie Colemeadow, Basim Hussein, Varvara Bashkirova, Kavya Pillai, Lucy Singh, Mehar Chawla

**Affiliations:** 1 Colorectal Surgery, North Middlesex University Hospital NHS Trust, London, GBR; 2 General Surgery, North Middlesex University Hospital NHS Trust, London, GBR

**Keywords:** target referral, two week wait, colorectal cancer, referral, surgery, target, cancer, colorectal

## Abstract

Aim

Since the introduction of the target referral system, there has been controversy about its value and whether it affected the short- and long-term outcomes of colorectal cancer surgeries. With contradicting results, this study highlights differences in personal and tumour characteristics, management differences, and outcomes in each referral pathway, including target pathway referrals for suspected cancers, emergency presentations, routine referrals, and incidentally discovered cancers during screening.

Methods

A retrospective study of colorectal cancer (CRC) patients operated on between January 1, 2010, and December 31, 2014, with records dating to the end of the five-year follow-up, was extracted anonymously from the database of CRC outcomes at the North Middlesex University Hospital NHS Trust, London. The total number of patients operated on through the four pathways was 176, with full records and competent follow-ups. Patients were classified according to the mode of referral: two-week wait (2WW or target), routine, emergency, and incidental discovery referrals. Comparisons were made between these groups with regard to personal and tumour characteristics, management, and outcome.

Results

It has been demonstrated by this study that target referrals present mainly with stage I cancers as compared to emergency referrals that present with more stage II (IIa+ IIb+ IIc). The highest percentage of cancer locations within the large bowel was rectal, followed by sigmoid in both target and emergency groups; 8.8% of target patients needed neoadjuvant chemoradiotherapy in the form of FOLFOX (folinic acid, 5-fluorouracil, and oxaliplatin) chemotherapy protocol with the addition of radiotherapy in patients with advanced rectal cancers, compared to 13.3% of emergency patients.

Conclusion

The colorectal 2WW system was the main pathway supplying colorectal cancer operations; it mostly showed earlier cancers than the other referral groups; its cancers were mostly rectosigmoid with less need for adjuvant chemotherapy; fewer recurrences; and it also showed a lower five-year mortality rate than the emergency group.

## Introduction

Colorectal cancer (CRC) is a global health burden, accounting for almost 700,000 deaths per year worldwide [1]. CRC is the third most commonly diagnosed cancer worldwide and second in Europe [1]. According to the World Health Organization's Global Cancer Observatory (GLOBOCAN) database, in 2012, almost 1.4 million new cases of CRC were diagnosed, and almost 700,000 deaths occurred worldwide [1]. It remains the second-most common cause of cancer death in the UK. The five-year survival rate is approximately 58%; however, this increases to over 90% if CRC is diagnosed in its earliest stage [2].

The risk of CRC rises with age, and its occurrence is uncommon before the fourth decade [1,3]. This is why most screening programs are aimed at people over the age of 50. Nonetheless, recent studies have found a worrisome increase in occurrence between the ages of 40 and 44, prompting some to suggest that the recommended screening age be lowered [4,5]. In contrast to impoverished portions of the world, where mortality rates are either stable or growing, most economically developed countries have seen a steady drop in mortality rates [1,6]. This reflects the wide range of screening options, specialized care, and lifestyle risk factors available [6]. Central-Eastern Europe has the highest recorded mortality rates, whereas Middle-Western Africa has the greatest incidence-to-fatality ratio [1,7].

Some cancer patients were facing severe delays in receiving outpatient appointments in secondary care, which could result in a delay in the diagnosis and progression of the disease [8]. In 2000, the Department of Health created the NHS Cancer Plan to make it easier to find a colorectal expert. The Two-Week Rule (TWR), which specifies that all units must see 95% of possible cancer patients, is part of the NHS Cancer Plan.

The TWR was created in part to ensure that patients with symptoms strongly suggestive of CRC were seen in secondary care within 14 days, allowing for timely examination and investigation. As a result, prompt treatment may be possible, lowering morbidity and death. For each form of cancer, a set of TWR referral criteria was developed. The TWR CRC referral criteria, published by the UK government in 2000, were created to help general practitioners (GPs) identify which patients deserved to be referred based on symptomatology that was linked to a high risk of CRC [8,9].

The National Institute for Health and Care Excellence (NICE) has established criteria for determining the sort of target referral to be made (2WW) [10].

The Scottish Intercollegiate Guidelines Network (SIGN) [11] recommends that patients with colorectal cancer wait no more than 62 days between their initial referral and the start of definitive treatment.

Despite the two-week wait (2WW) referral pathway, a significant proportion of patients with new bowel symptoms are still referred via the other pathways, including the ‘emergency’ and ‘routine’ referral pathways. The national bowel cancer screening program (NBCSP) and screening of high-risk groups (family history and inflammatory bowel disease) have been shown to detect early asymptomatic cancers (incidental discoveries) with improved disease-specific survival.

This study aims to compare the above-mentioned four pathways of referral with regard to patient statistics: sex, tumour location and stage, type of operation, need for a stoma, postoperative leak, need for chemotherapy, and five-year mortality.

## Materials and methods

The North Middlesex University Hospital (NMUH) NHS Trust diagnoses and treats a significant number of new CRC cases every year, and all patients are treated according to national guidelines. In addition to the "2WW", symptomatic patients are referred by general practitioners using the "routine" referral pathway for outpatient consultation, and by using "emergency" referral to the surgical admission unit, in conjunction with the National Bowel Cancer Screening Programme (NBCSP), the colorectal department receives cases of bowel cancer discovered by bowel cancer screening centres. The database of CRC outcomes at NMUH Trust was analysed anonymously.

A retrospective study was conducted on the data available for patients operated on for CRC between January 2010 and December 2014; data analysis was done according to the mode of presentation.

The total number of patients operated on through the four pathways was 198; patients with incomplete records or lost from the five-year follow-ups were excluded, leaving 176 patients with full records and completed five years of follow-ups, unless death had occurred.

Comparisons were made between the different referral groups (2WW, routine, emergency, and incidental discovery during screening program or investigation for another medical condition); comparisons were made with regards to the number of patients included in each referral type, sex distribution, location of the colorectal cancers discovered, cancer stage at the time of initial diagnosis, type of operation done (laparoscopic, open, or converted), need for a stoma, need for chemotherapy, anastomotic leak rate, management of leak, recurrence rates, and finally, mortality rates within five years of the initial diagnosis.

## Results

Over five years, starting on January 1, 2010, and ending on December 31, 2014, a total of 176 patients were operated on for colorectal cancers in North Middlesex University Hospital NHS Trust, London, as per electronic colorectal cancer records provided anonymously by the informatics department. They were also followed up for a period of five years, unless death had occurred during this period of follow-up.

**Figure 1:**
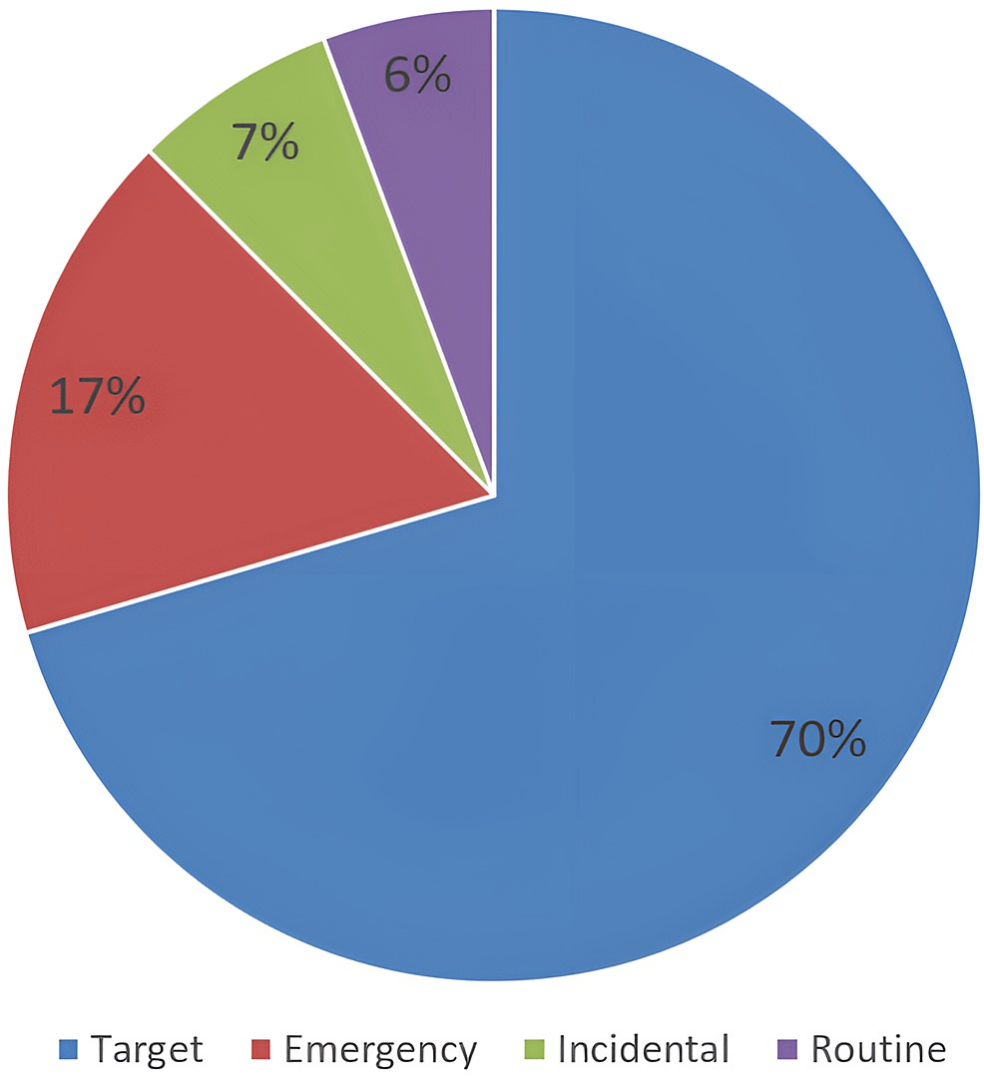
Percentage of patients by referral type

Out of these 176 patients, there were 124 target referrals seen within two weeks of referral, and there were 30 emergency referrals, where patients were admitted for rectal (PR) bleeding, bowel obstruction, and abdominal pain and were diagnosed and managed for colorectal cancer operatively following this admission. There were also 10 routine GP referrals that turned out to be cancers and were managed operatively too. The last group was when the CRC was discovered incidentally during routine National Bowel Cancer Screening Program (NBCSP) or high-risk group screening, such as in cases of inflammatory bowel disease or sometimes during CT scanning for a different non-bowel-related condition; this group comprised 12 patients (Figure 1).

The gender distribution in this study was nearly equal (87 males (M) to 89 females (F)), with a higher female number in the target (65F, 59M) and emergency (17F, 13M) groups and a male predominance in the routine referral (8M, 2F) and incidental (7M, 5F) groups.

**Figure 2:**
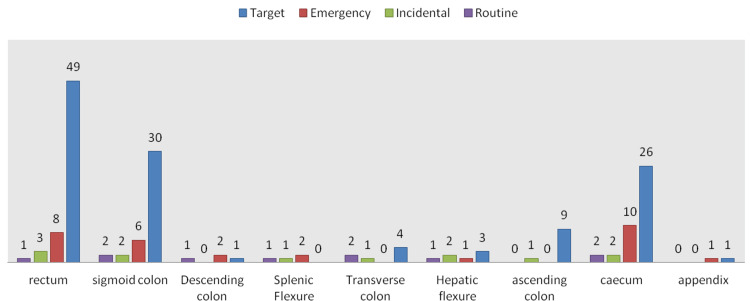
Cancer locations according to the type of referral

The highest percentage of cancer locations within the large bowel was rectal in the target referral group (39.5%), followed by the sigmoid colon (24.1%). Emergency pathway cancers followed the same mode of distribution, where the highest incidence was in the rectum (26.6%), followed by the sigmoid colon (20%), whereas routine referral cancers showed a shared top rank between caecal, transverse colon, and sigmoid, each representing 20% of the total, while incidental discovery showed a quarter of the cancers occurring in the rectum and one-sixth occurring in the sigmoid colon (Figure 2).

This study explored the stage of colorectal cancer at diagnosis. It was found that the highest percentage of target patients were at stage I upon diagnosis (53 patients out of 124 target referrals, 42.7%), while most of the operated emergency group belonged to stage II (IIa: four, IIb: six, and IIc: three), with a total of 13 patients out of 30 (43.3%), while in routine referrals, five out of 10 belonged to stage III (50%), while the incidental discovery of cancers yielded 50% of the cancers to be at stage I upon diagnosis.

It has been proven by this study that target referrals present mainly with stage I cancers as compared to emergency referrals that present with more stage II (IIa+IIb+ IIc) with a relative risk of 2.589 and a confidence interval from 1.081 to 6.201 (Tables 1-2).

**Table 1:** Comparison between the different studied groups of referral according to cancer stage at diagnosis χ2: Chi-square test; MC: Monte Carlo; p: p-value for comparing the studied groups; *: statistically significant at p ≤ 0.05

Cancer stage at diagnosis			Type of referral						χ2	MC p
	Target (n = 124)		Emergency (n = 30)		Incidental (n = 12)		Routine (n = 10)			
	No.	%	No.	%	No.	%	No.	%		
I	53	42.7	4	13.3	6	50.0	2	20.0	11.558^*^	0.006^*^
IIa	13	10.5	4	13.3	2	16.7	1	10.0	1.097	0.827
IIb	11	8.9	6	20.0	1	8.3	1	10.0	3.160	0.296
IIc	10	8.1	3	10.0	1	8.3	0	0.0	0.781	0.904
IIIa	12	9.7	1	3.3	1	8.3	2	20.0	2.835	0.354
IIIb	8	6.5	8	26.7	1	8.3	2	20.0	10.250^*^	0.009^*^
IIIc	11	8.9	1	3.3	0	0.0	1	10.0	1.579	0.577
IVa	3	2.4	1	3.3	0	0.0	0	0.0	0.917	0.747
IVb	2	1.6	0	0.0	0	0.0	0	0.0	1.520	1.000
IVc	1	0.8	2	6.7	0	0.0	1	10.0	6.612	0.083
χ2 (MCp)			34.119^* ^(0.046^*^)							

**Table 2:** Comparison between emergency and target referral groups according to the four main cancer stages at diagnosis The relative risk is 2.589, and the confidence interval is from 1.081 to 6.201, according to the theory that target referrals present mainly with stage I as compared to emergency referrals that present with more stage II (IIa+ IIb+ IIc).

Cancer stage at diagnosis	Emergency	Target
II (IIa +IIb +IIc)	13	34
III (IIIa +IIIb +IIIc)	10	31
IV (IVa +IVb +IVc)	3	6
I	4	53

Seventy-three percent of the cancers in the target group were operated on successfully with laparoscopy. The conversion rate to open procedure was 14.5%; in emergency referrals, there was an equal distribution of cases operated on laparoscopically or through open surgery from the start, with 13 cases out of 30 (43.3%) each. There was no need to start any case as an open procedure in the incidental discovery group, and two patients out of 12 needed conversion intraoperatively; 70% of routine referrals could still be done successfully laparoscopically.

It was statistically concluded from this study that target referral-type patients’ operations were done laparoscopically more than emergency referral-type patients' operations, and that emergency referral-type patients had more operations started as open procedures than target referral-type patients, with a relative risk of 1.717 and confidence intervals from 1.160 to 2.541 (Tables 3-4).

**Table 3:** Comparison between the different studied groups according to the type of referral and type of operative intervention χ2: Chi-square test; MC: Monte Carlo; p: p-value for comparing the studied groups; *: statistically significant at p ≤ 0.05

Type of operative intervention			Type of referral						χ2	MC p
	Target (n = 124)		Emergency (n = 30)		Incidental (n = 12)		Routine (n = 10)			
	No.	%	No.	%	No.	%	No.	%		
The operation was done laparoscopically	91	73.4	13	43.3	10	83.3	7	70.0	10.600^*^	0.010^*^
The operation started as an open procedure	15	12.1	13	43.3	0	0.0	1	10.0	16.233^*^	0.001^*^
The operation was converted from laparoscopic to an open surgery	18	14.5	4	13.3	2	16.7	2	20.0	0.745	0.889
χ2 (MCp)			17.256^* ^(0.005^*^)							

**Table 4:** Comparison between emergency and target referrals according to the type of operative intervention The relative risk is 1.717, and the confidence interval is from 1.160 to 2.541 for the theory that target referral type patients’ operations were done laparoscopically more than emergency patients and that emergency referral type patients had more operations started as open procedures than target referral type patients.

Type of operative intervention	Type of referral emergency (n = 30)	Target (n = 124)
The operation started as an open procedure	13	15
The operation was done laparoscopically	13	91

**Figure 3:**
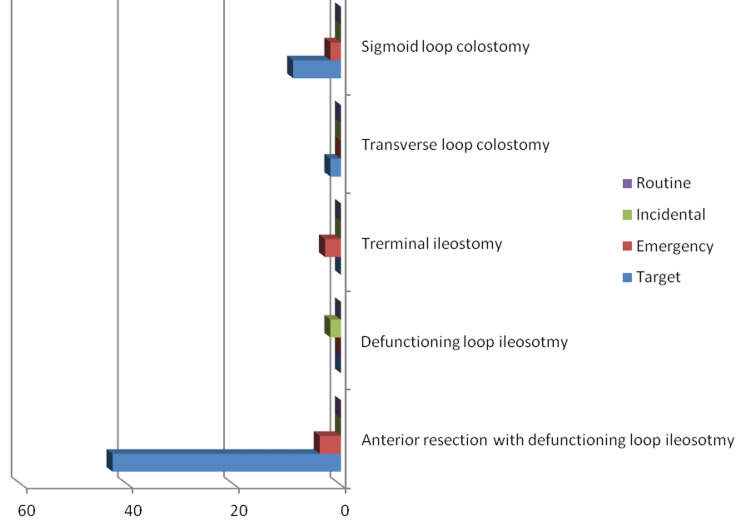
Types of stomas created for different types of referral

Excluding the 43 patients with low rectal cancers who needed a defunctioning (diverting) loop ileostomy as part of their surgery, whether open or laparoscopically, two transverse and nine sigmoid loop colostomies were needed as surgical management for target referral patients, while three terminal ileostomies and two sigmoid colostomies were needed for the management of five patients out of 30 emergency referrals. We could also see that only two defunctioning loop ileostomies were needed for all of the incidental finding groups, and no stomas were needed for any of the routine referral cancers (Figure 3).

**Figure 4:**
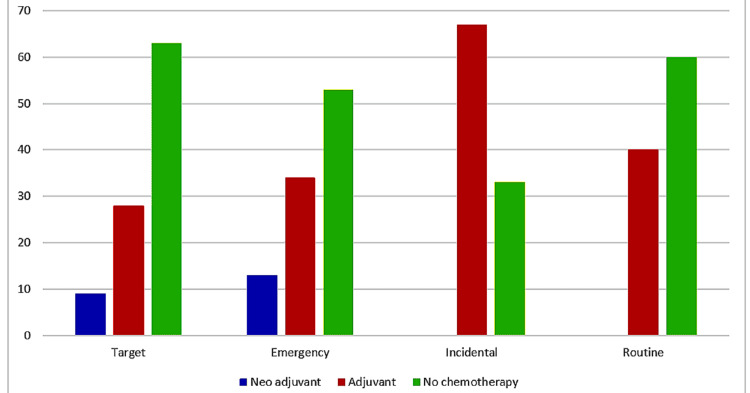
Percentage of patients' need for chemotherapy per referral type

Most of the patients in each group did not need chemotherapy; 8.8% of target patients needed neoadjuvant chemotherapy in the form of the FOLFOX (folinic acid, 5-fluorouracil, and oxaliplatin) chemotherapy protocol, with the addition of radiotherapy in patients with advanced rectal cancers, compared to 13.3% of emergency patients; neither incidental nor routine patients needed any neoadjuvant treatment; around one-fourth of target patients needed adjuvant chemotherapy; while one-third of emergency referrals and three-fourths of the incidental group were candidates for chemotherapy. Forty percent of routine referrals were sent for adjuvant treatment (Figure 4).

Regarding the anastomotic leaks in the target group, there were two: one after laparoscopic anterior resection of the cancer rectum without ileostomy, managed conservatively; the other, after laparoscopic sigmoid colectomy, had laparotomy for the management of the leak. While in the emergency group, there were also two leaks, both after laparoscopic right hemicolectomy; both had laparotomies; one had an end-ileostomy; the other had a refashioned anastomosis; no leaks were encountered for the incidental or routine referrals.

Thirty-nine out of 124 target patients (31.4%) sadly passed away during the five-year follow-up interval, while 14 out of 30 emergency patients (46.6%) were not alive by the end of the five-year period. Two out of 12 (16.6%) incidental finding patients and also two out of 10 (20%) routine referral patients lost their lives before the end of the five years since diagnosis.

There were 11 documented recurrences of cancer in the target group (8.8%), with five of these recurring as hepatic deposits; eight of the emergency referral patients had recurrences (26.6%); five of these were lung deposits; two recurrences were noted in the incidental group (16.6%), while a 10% recurrence rate was recorded for the routine referral group.

Mortality within five years of operation was studied, and the results showed that the highest percentage recorded postoperatively in the emergency referral group was 46.6%, while the target group showed 31.4%, the routine referral group came in third with 20%, and the incidental finding group had a 16.6% mortality rate within five years of surgery.

## Discussion

The 2WW referral system was developed to ensure that patients with symptoms that could indicate colorectal cancer were assessed and evaluated by a colorectal specialist within 14 days, allowing for faster diagnosis and treatment. Most of the colorectal cancer patients operated on in the current study were referred via the 2WW target referral pathway (Figure 1), and the majority of the colorectal cancers referred via the target pathway were rectosigmoid, followed by caecal cancers (Figure 2).

A retrospective study of 462 patients seen in the fast-track clinic was conducted over an 18-month period. Patients who were referred to the fast-track clinic were seen sooner than those who were referred via a standard letter, but they had more advanced disease according to Duke's staging system [9]. However, this study comprised operative and non-operative patients, while the current study focused only on operated patients; this can explain the dissimilarity of the findings, as in the current study, most of the target referral’s operated patients were stage I upon presentation, while most of the routine referral’s operated patients’ were stage III (Tables 1, 2).

In the current study, the rate of laparoscopic conversion to open was 14.5% for the target group, 23.3% for the emergency group, 16.6% for the incidental finding group, and 22.2% for the routine referral group; these results are close to a study by Francis et al. [12], which showed a single surgeon conversion rate of 20.4%. A two-step cluster analysis identified 406 patients in cluster I (lower risk), with a conversion rate of 8%, and 261 patients in cluster II (higher risk), with a conversion rate of 30%.

According to Boccola et al. [13], anastomotic leak rates after colorectal anastomosis ranged from 4% to 26%, and the development of a leak has been linked to a worse prognosis following colon cancer curative resections. In the current study, there were two target referral patients (2.8%) and two emergency patients (9.5%) with anastomotic leaks out of 111 patients who had anastomoses (3.6%), which is a very low rate as compared to other studies of colorectal cancer complications.

The current study showed that the five-year mortality in the target group was less than that in the emergency group but higher than that in the incidental discovery and routine referral groups (31.4%, 46.6%, 16.6%, and 20%, respectively).

Ramos et al. [14] did a comprehensive analysis of Medline, Embase, Cancerlit, and the Cochrane Database of Systematic Reviews to find papers dealing with delays in diagnosis and treatment in relation to survival in patients with colon cancer that were published between 1962 and 2006. The result of a meta-analysis based on the calculation of the relative risk (RR) and a model of random effects suggests that in colorectal cancer patients, there is no link between diagnostic and treatment delays and survival. The colon and rectum should be evaluated separately, and other relevant variables such as tumor stage should be taken into account.

Patel et al. [11] studied, between January 1999 and June 2005, 1,012 patients who were treated for colorectal cancer and divided them into four groups: standard met (elective), standard met (emergency), standard failed (elective), and standard failed (emergency). They concluded that patients who were not treated within the time frame set by the SIGN guidelines lived longer after surgery. This is most likely due to a combination of factors, including the pathological cancer stage.

In a study by Walsh et al. [15], a prospective database was used to identify patients diagnosed with colorectal cancer before (group 1) and after (group 2) the implementation of the two-week rule. After excluding emergency patients, this study concluded that there has been no improvement in two-year survival from colorectal cancer since the implementation of the two-week rule, while Zafar et al. [16] found that the five-year survival rates in the pre-2WW and post-2WW groups did not differ significantly (71% vs. 72%, respectively; p=0.880).

Similar to the study by Schneider et al. [17], they found that out of 189 patients, 96 (51% of them) presented via the fast track (FT), 41 (22.5%) via the non-fast track (NFT), and 52 (27.5%) via the emergency room (ER) referral route. Five-year overall survival and cancer-specific survival were studied; patients referred to as emergencies had worse five-year overall survival, and differences in five-year survival did not reach statistical significance in these patients.

Mangion et al. reviewed the literature [18]. The goal of this literature study was to look at the link between the "two-week rule" (TWR) and colorectal cancer diagnosis (CRC). After exploring the Cochrane Review, the Cumulative Index to Nursing and Allied Health Literature (CINAHL), the NHS Evidence, and the Medline databases, twelve papers were discovered. Since the TWR's introduction, the CRC detection rate has been poor, and there appears to be disagreement in the literature about the TWR's efficacy in detecting CRC at an earlier stage.

Limitations

It is worth mentioning here that there can be some limiting factors to the type of the current study; these may include factors related to cancer itself, such as the early presentation of some cancers versus the late presentation of others, which may depend on cancer aggression and location. Other factors might be patient-related, including age, gender, socio-economic status, and ethnicity, which could influence referral patterns.

## Conclusions

The colorectal 2WW system was the main pathway supplying colorectal cancer operations to North Middlesex Hospital during the study period; it mostly helped detect cancers earlier than the other referral groups. The cancers detected were mostly rectosigmoid, with less need for adjuvant chemotherapy, fewer recurrences, and a lower five-year mortality rate than the emergency group.

In summary, and as seen from this study, the target (2WW) referral constitutes a major part of the colorectal cancer management system and has affected it positively in multiple aspects.
